# Comparison of bilateral decompression via unilateral laminotomy and conventional laminectomy for single-level degenerative lumbar spinal stenosis regarding low back pain, functional outcome, and quality of life - A Randomized Controlled, Prospective Trial

**DOI:** 10.1186/s13018-019-1298-3

**Published:** 2019-08-08

**Authors:** Sangbong Ko, Taebum Oh

**Affiliations:** Department of Orthopaedic Surgery, College of Medicine, Daegu Catholic University, 33, Duryugongwon-ro 17-gil, Nam-gu, Daegu, 42472 South Korea

**Keywords:** Lumbar spine, Spinal stenosis, Decompression, Low back pain

## Abstract

**Background:**

Conventional posterior open lumbar surgery is associated with considerable trauma to the paraspinal muscles. Severe damage to the paraspinal muscles could cause low back pain (LBP), resulting in poor functional outcomes. Thus, several studies have proposed numerous surgical techniques that can minimize damage to the paraspinal muscles, particularly unilateral laminotomy for bilateral decompression. The purpose of this study is to compare the degree of postoperative LBP, functional outcome, and quality of life of patients between bilateral decompression via unilateral laminotomy (BDUL; group U) and conventional laminectomy (CL; group C).

**Methods:**

Of 87 patients who underwent diagnostic and decompression surgery, 50 patients who met the inclusion and exclusion criteria and were followed up for > 2 years were enrolled. The patients were asked to record their visual analog scale pain score after 6, 12, and 24 months postoperatively. BDUL was used for group U, whereas CL was used for group C. The patients were randomly divided based on one of the two techniques, and they were followed up for over 2 years. Functional outcomes were assessed by the Oswestry Disability Index (ODI), Roland–Morris Disability Questionnaire (RMDQ), and SF-36.

**Results:**

Operation time was significantly shorter in group U than in group C (*p* = 0.003). At 6, 12, and 24 months, there was no significant difference between the two groups in terms of spine-related pain (all *p* > 0.05). Functional outcomes using ODI and RMDQ and quality of life using SF-36 were not significantly different between the groups (all *p* > 0.05).

**Conclusions:**

Regarding single-level decompression for degenerative lumbar spinal stenosis, group U had the advantages of shorter operation time than group C, but not in terms of back pain, functional outcome, and quality of life.

## Introduction

In the absence of any improvement or deterioration following diverse conservative treatments or in the presence of neurological deficit, surgical treatment is warranted for degenerative lumbar spinal stenosis (DLSS). Typically, surgery is performed using a posterior approach for adequate decompression of the stenotic site. Conventional laminectomy (CL) is usually performed for adequate decompression, but it can damage the posterior structures that provide lumbar stability during flexion. In addition, excessive intraoperative resection of the facet joints could result in postoperative segmental instability, which could necessitate additional fusion surgery [1]. Moreover, conventional posterior open lumbar surgery is associated with considerable trauma to the paraspinal muscles. Even without substantial damage to the facet joints, severe damage to the paraspinal muscles could cause low back pain (LBP), resulting in poor functional outcomes. Some recent studies have suggested a correlation of atrophy of the multifidus muscle and chronic LBP with poor circulation of arterial blood, excessive muscle stripping, and damage to the posterior branches of dorsal rami following prolonged intraoperative muscle traction [2–11].

Thus, several studies have proposed numerous surgical techniques that can minimize damage to the paraspinal muscles, particularly unilateral laminotomy for bilateral decompression [12, 13]. In addition, these studies have assumed that bilateral decompression via unilateral laminotomy (BDUL) can postoperatively decrease damage to soft tissues and the degree of LBP compared with CL. These findings suggest that postoperative functional outcome and quality of life of patients is enhanced following BDUL.

Hence, this study aimed to compare the degree of postoperative LBP, functional outcome, and quality of life of patients between BDUL (group U) and CL (group C), which were randomly performed by a spine surgeon in patients with single-level DLSS requiring surgery.

## Materials and methods

### Study design and patient selection

**Fig. 1:**
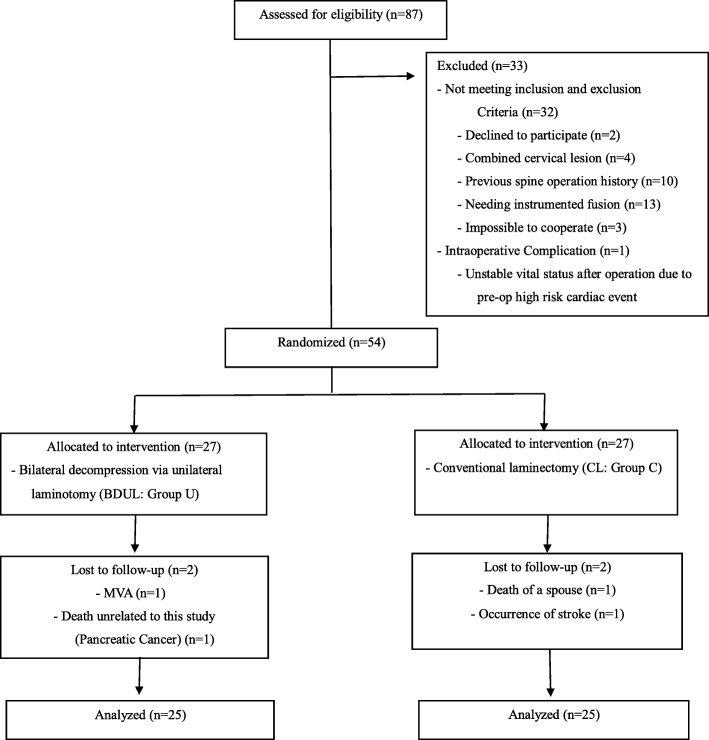
Flow diagram showing the procedure used in this study

Among 87 patients who underwent diagnostic and decompression surgery from 1 January 2015 to 30 June 2016, 50 patients who met the inclusion and exclusion criteria (Table 1) and who were followed up for > 2 years were prospectively analyzed in this study (Fig. 1). The surgical indication of patients is not LBP but is neurogenic claudication with radiating pain. The between-group difference in terms of SF-36 at postoperative 1 year was 3.2 (standard deviation, 3.5); we needed a sample size of 25 patients in each group to yield a power of 89% with a significance level of 0.025 (two pairwise comparisons) [14]. Thus, we required sample size of a total of 54 patients in this study, assuming a 10% dropout rate. Randomization was based on the CONSORT guidelines, and we categorized subjects eligible for selection into two groups (groups C and U) at a ratio of 1:1. Furthermore, random selection was determined using the RANDBETWEEN function of Microsoft Excel version 14.0 for Windows.

**Table 1:** Inclusion and exclusion criteria

Inclusion criteria	
1	Patients with degenerative lumbar spinal stenosis requiring surgery due to neurogenic claudication with radiculopathy
2	Patients with one-level central stenosis requiring decompression
3	Patients with MRI findings consistent with symptoms on preoperative radiological examination
4	Patients who agreed to this study
Exclusion criteria	
1	Patients who underwent spinal surgery in the past
2	In addition to decompression, patients who needed further segmental fusion surgery.
3	Patients who require multiple segments of decompression surgery( ≥ 3 levels )
4	Patients with cervical lesions other than lumbar lesions.
5	Patient with rapidly progressive neurological deficit
6	Patients who cannot cooperate in completing the questionnaire due to dementia or stroke
7	Neuromuscular disorder
8	Spinal malignancy, spinal infection, etc.

### Surgical technique

#### CL (group C)

The lumbosacral fascia was dissected along the midline, and the paraspinal muscles of bilateral sides were stripped from the spinous process and lamina using electrocautery or Cobb’s elevator. Using a Gelpi self-retractor, the soft tissue was pulled to expose the medial side of the bilateral facet joint. After resection of the spinous process, the cranial one-third of the lower lamina and caudal two-thirds of the upper lamina were resected with a burr and Kerrison rongeur, followed by the ligamentum flavum and medial one-third of the inferior articular process, and surgeons confirmed that the dura and nerve root were not compressed.

#### BDUL (group U)

BDUL was performed based on the surgical technique described by Spetzger et al. [12]. The side of approach was decided by surgeons’ preference, with the right side being the most common side. Using a surgical microscope, the lumbosacral fascia was dissected along the midline, and the paraspinal muscles of the unilateral side were stripped without any damage to the supraspinous and interspinous ligaments and the paraspinal muscles of the other side. Then, the cranial and caudal portions of the unilateral lamina, ligamentum flavum, and medial side of the facet joint were exposed. Next, unilateral paraspinal muscles were pulled with a Gelpi self-retractor. The lamina bone from two-thirds of the cranial lamina to one-third of the caudal lamina and one-third of the medial facet joint were resected with a burr and Kerrison rongeur. After establishing that the dural and nerve roots were not compressed, the operating table was inclined at 30°–40° and the microscope was directed toward the other side to observe the opposite ligamentum flavum and nerve root without undercutting the base of the spinous process. The dissector was used to eliminate the medial side of the ligamentum flavum and the superior articular process while protecting the dural and contralateral nerve roots. Based on operative findings, only the contralateral ligament was removed in severe cases of ligamentum flavum hypertrophy.

#### Postoperative care

In this study, a discectomy was not performed in all cases, and surgery was performed by one spine surgeon. Both groups were recommended to walk after wearing a corset on the first postoperative day and were discharged within 5–7 days after surgery. The corset was worn for 2–4 weeks with persistent LBP, and isokinetic back muscle exercise was performed 7 days after surgery if postoperative LBP was tolerable to minimize back muscle atrophy.

### Radiological and outcome measurement

Preoperative MRI of all patients showed bilateral central stenosis alone without foraminal stenosis. Postoperative radiological assessment was performed after 6, 12, and 24 months. Patients confirmed having instability when > 4-mm translation or > 10° angulation was viewed in lateral flexion and extension images. We used the visual analog scale (VAS; 0–10) to assess the degree of LBP preoperatively as well as at 6, 12, and 24 months postoperatively. The functional outcome was evaluated after 6, 12, and 24 months using the Oswestry Disability Index (ODI) and Roland–Morris Disability Questionnaire (RMDQ). In addition, a researcher not involved in this study measured the quality of life of patients using the SF-36 physical component scale (PCS) and mental component scale (MCS) at 6, 12, and 24 months postoperatively.

### Statistical analysis

In this study, all statistical analyses were performed with SPSS version 19.0 for Windows (SPSS, IBM Corporation). We considered *p* ≤ 0.05 as statistically significant. In addition, between-group differences in terms of VAS scores and functional outcomes were analyzed using Mann–Whitney *U* test. Notably, the power of result in the VAS score difference and functional outcome was determined with G power 3.1. In the event of statistical significance, we performed unpaired *t* test. The level of significance was set at *p* < 0.05.

## Results

### Epidemiological and radiological results

The mean age of the 50 study patients was 67.16 ± 9.45 (range 38–81) years; it was 66.24 ± 8.11 (range 40–78) years in group C and 68.08 ± 10.72 (38–81) years in group U, with no significant difference in terms of age (*p* = 0.49). While group C comprised 10 males and 15 females, group U had 8 males and 17 females. Preoperative LBP was 5.20 ± 2.843 in group C and 4.20 ± 2.198 in group U, preoperative referred buttock pain was 5.20 ± 2.958 in group C and 4.56 ± 3.292 in group U, and preoperative radiating lower leg pain was 7.20 ± 1.633 in group C and 7.28 ± 1.221 in group U. We observed no significant difference between the two groups in terms of all types of pain (*p* = 0.171, 0.473, and 0.697, respectively). In group C, there was 1 patient with pain at L3–4 level, 19 patients at L4–5 level, and 5 patients at L5–S1 level; in group U, there was 1 patient with pain at L3–4 level, 18 patients at L4–5 level, and 6 patients at L5–S1 level. We noted no cases of instability in the operative segment and adjacent segments on simple radiographs at 24 months postoperatively. In addition, we observed no significant differences in terms of weight (*p* = 0.708) and height (*p* = 0.781) between the groups. Total hospitalization days were 5.67 ± 3.006 days in group C and 5.90 ± 3.632 days in group U (*p* = 0.818). Operation time was 83.81 ± 31.344 min in group C and 119.38 ± 39.798 min in group U. Furthermore, operation time was significantly shorter in group U than in group C (*p* = 0.003; Table 2).

**Table 2:** Demographics of all population

		Group C	Group U	*p* value
Total population		25	25	
Male/female		10/15	8/17	
Mean age (years)		66.24 ± 8.110	68.08 ± 10.716	0.49
Adm. date		5.67 ± 3.006	5.90 ± 3.632	0.818
OP time (min)		83.81 ± 31.344	119.38 ± 39.798	0.003
Body weight (kg)		58.38 ± 9.222	59.62 ± 11.885	0.708
Height (cm)		156.33 ± 7.010	157.10 ± 10.334	0.781
Affected levels	L3–4	1	1	
	L4–5	19	18	
	L5–1	5	6	

### Clinical symptoms

LBP after 6 months was 3.12 ± 1.943 in group C and 2.98 ± 2.267 in group U, referred buttock pain was 3.08 ± 2.290 in group C and 2.74 ± 2.380 in group U, and radiating lower leg pain was 2.76 ± 1.964 in group C and 2.82 ± 2.147 in group U; we observed no significant difference between the groups in terms of all types of pain (*p* = 0.641, 0.308, and 0.838, respectively). LBP after 12 months was 2.16 ± 1.599 in group C and 2.02 ± 1.269 in group U, referred buttock pain was 2.24 ± 1.809 in group C and 1.80 ± 1.573 in group U, and radiating lower leg pain was 1.76 ± 1.985 in group C and 1.80 ± 1.573 in group U; we observed no significant difference between the groups in terms of all types of pain (*p* = 0.496, 0.806, and 0.875, respectively). Further, LBP after 24 months was 1.56 ± 1.734 in group C and 1.38 ± 1.190 in group U, referred buttock pain was 1.08 ± 1.631 in group C and 1.04 ± 1.080 in group U, and radiating lower leg pain was 1.04 ± 1.744 in group C and 1.20 ± 1.150 in group U; we observed no significant difference between the groups in terms of all types of pain (*p* = 0.396, 0.839, and 0.447, respectively; Table 3).

**Table 3:** The results of spine-related pain

		Group C	Group U	*p* value
Initial	Back	5.20 ± 2.843	4.20 ± 2.198	0.171
	Buttock	5.20 ± 2.958	4.56 ± 3.292	0.473
	Leg	7.20 ± 1.633	7.28 ± 1.221	0.697
6 months	Back	3.12 ± 1.943	2.98 ± 2.267	0.641
	Buttock	3.08 ± 2.290	2.74 ± 2.380	0.308
	Leg	2.76 ± 1.964	2.82 ± 2.147	0.838
12 months	Back	2.16 ± 1.599	2.02 ± 1.269	0.496
	Buttock	2.24 ± 1.809	2.18 ± 1.616	0.806
	Leg	1.76 ± 1.985	1.80 ± 1.573	0.875
24 months	Back	1.56 ± 1.734	1.38 ± 1.190	0.396
	Buttock	1.08 ± 1.631	1.04 ± 1.080	0.839
	Leg	1.04 ± 1.744	1.20 ± 1.150	0.447

### Functional outcome

At 6 months postoperatively, ODI was 16.16 ± 6.101 and RMDQ was 8.32 ± 5.475 in group C and 15.92 ± 9.668 and 7.74 ± 6.342, respectively, in group U; we observed no significant difference between the two groups in terms of functional outcome after 6 months (*p* = 0.835 and 0.492, respectively). At 12 months postoperatively, ODI was 15.04 ± 9.432 and RMDQ was 7.52 ± 7.218 in group C and 15.00 ± 7.887 and 6.70 ± 4.362, respectively, in group U; we observed no significant difference between the groups in terms of functional outcome after 12 months (*p* = 0.974 and 0.336, respectively). At 24 months postoperatively, ODI was 11.44 ± 6.740 and RMDQ was 4.68 ± 5.289 in group C and 11.96 ± 8.166 and 4.60 ± 3.618, respectively, in group U; we observed no significant difference between the groups in terms of functional outcome after 24 months (*p* = 0.626 and 0.901; Table 4). SF-36 PCS was 41.91 ± 25.604 after 6 months, 50.10 ± 22.318 after 12 months, and 65.34 ± 23.177 after 24 months in group C and was 40.24 ± 22.030 after months, 47.61 ± 17.284 after 12 months, and 63.96 ± 20.146 after 24 months in group U; we observed no significant difference between the two groups in terms of time (*p* = 0.622, 0.383, and 0.655, respectively). SF-36 MCS was 50.03 ± 22.494 after 6 months, 60.56 ± 19.823 after 12 months, and 73.70 ± 18.845 after 24 months in group C and was 47.18 ± 19.669 after 6 months, 56.87 ± 23.391 after 12 months, and 70.36 ± 18.310 after 24 months in group U. We observed no significant difference between the groups in terms of time (*p* = 0.345, 0.235, and 0.210, respectively; Table 5).

**Table 4:** The result of functional outcome

		Group C	Group U	*p* value
6 months	ODI	16.16 ± 6.101	15.92 ± 9.668	0.835
	RMDQ	8.32 ± 5.475	7.74 ± 6.342	0.492
12 months	ODI	15.04 ± 9.432	15.00 ± 7.887	0.974
	RMDQ	7.52 ± 7.218	6.70 ± 4.362	0.336
24 months	ODI	11.44 ± 6.740	11.96 ± 8.166	0.626
	RMDQ	4.68 ± 5.289	4.60 ± 3.618	0.901

**Table 5:** Result of quality of life using SF-36 questionnaire

		Group C	Group U	*p* value
6 months	SF-36 PCS	41.91 ± 25.604	40.24 ± 22.030	0.622
	SF-36 MCS	50.03 ± 22.494	47.18 ± 19.669	0.345
12 months	SF-36 PCS	50.10 ± 22.318	47.61 ± 17.284	0.383
	SF-36 MCS	60.56 ± 19.823	56.87 ± 23.391	0.235
24 months	SF-36 PCS	63.34 ± 23.177	63.96 ± 20.146	0.655
	SF-36 MCS	73.70 ± 18.845	70.36 ± 18.310	0.210

## Discussion

In DLSS, the compression of a nerve (cauda equina and nerve root) by degenerated and hypertrophied bony structure and surrounding soft tissue causes symptoms such as LBP, referred buttock pain, radiating lower leg pain, and intermittent neurogenic claudication. The primary surgical treatment for this condition involves adequate decompression of the nerve, and the leading treatment failure in decompression surgery is inadequate decompression. Of note, segmental fusion should be performed if the posterior stabilizing structures are damaged and followed by the occurrence of segmental instability [15]. To date, several studies have reported adequate decompression to be the most successful surgical option for DLSS alone. Reportedly, the incidence of complications increases with the use of combined decompression and fusion surgery, and there is no evidence that the outcomes are superior to decompression alone [16, 17]. In addition, CL for central DLSS is a method that involves dissection of the posterior paraspinal muscles of bilateral sides as well as of the spinous process and supraspinous and interspinous ligaments. However, this posterior structure plays a crucial role in lumbar stability during lumbar flexion, and its excessive resection might cause LBP; therefore, it is essential to minimize its damage as much as possible. CL and decompression can attain satisfactory neurological recovery, but it is inevitable that the multifidus is stripped during surgery and damaged during prolonged traction. While performing these procedures with the bilateral approach, muscle damage is severer than that with the unilateral approach [12]. Lie et al. [18] reported that lumbar spinous process-splitting laminectomy decreased muscle damage and multifidus atrophy, leading to decreased LBP. Furthermore, several studies have reported BDUL to be a good treatment method in terms of morphological evaluation and postoperative spinal instability as well as symptom recovery [12, 19].

Nevertheless, Lie et al. [11] questioned the impact of LBP reduction because BDUL could damage the multifidus muscle in one lamina and the spinous process. Arai et al. [20] reported better results by unilateral approach than that by the bilateral approach but observed no difference between the two groups at a single level. However, multilevel LSS revealed a large difference in terms of back pain and functional outcome. The lack of statistical significance for LBP, functional outcome, and quality of life in both groups could be attributed to the fact that we performed single-segmental decompression using a microscope.

In this study, we observed no differences in terms of LBP, referred buttock pain, functional outcome, and quality of life between the groups after 6, 12, and 24 months because of short muscle traction time during surgery. Gejo et al. [21] reported that the traction of the paraspinal muscles for > 80 min (40–125 min) is only approximately 50% of the total muscle strength at 6 months postoperatively and that weakness of these muscles could cause LBP. We believe that although the total operation time of groups C and U was 83 and 119 min, respectively, from skin incision to skin closure, the muscle retraction time in both groups was < 80 min. Moreover, we thought that the use of intermittent Gelpi retractor instead of continuous Beckman retractor could decrease damage to the posterior muscle structures. On postoperative day 7, the trained back muscle isokinetic exercise was performed by the medical staff to help decrease LBP and recover the trunk muscle performance gradually [21]. In a study on adolescents, Remes et al. [22] reported that LBP was severe at 3–6 months postoperatively and did not markedly differ after 1 year of surgery and that motion preservation possibly plays a vital role in the maintenance of back muscle integrity, besides initial surgical trauma.

To date, few comparative studies have been conducted on single-level DLSS requiring decompression surgery. Fan et al. [23] reported a decline in postoperative LBP after a minimal level of multifidus and functional outcome in a minimal approach using a tubular system in single-level posterolateral interbody fusion surgery. This study comprised patients with lumbar spinal stenosis accompanied by instability and those who underwent fixation rather than decompression. The average operation time was 200 min, and retractor time was longer. In addition, Fan et al. [23] suggested that after 6 months of spinal surgery, the VAS score of LBP improved and ODI improved by the recovery process of the back muscle. Hong et al. [19] reported BDUL reduced the risk of late instability when compared with a conventional laminotomy. Although not a comparative study of the two surgical methods, Oertel et al. [13] reported that BDUL is an adequate technique for decompression of LSS in experienced hand. Dohzono et al. [24] reported microscopic BDUL prevents postoperative spinal instability. All of these studies included studies involving more than two levels, and no studies have analyzed only one level.

The present study has some limitations. One limitation is the lack of imaging findings, such as intramuscular pressure, paraspinal muscle edema in muscle damage, and pelvic parameter [25]; laboratory tests, such as serum creatine phosphokinase and inflammatory cytokine levels; and blood loss and transfusion volume during surgery. In addition, no radiological instability or increased severity of the LBP occurred at a mean of 24 months; however, the follow-up period was shorter to detect radiological changes and LBP. Despite the RCT study design, I could not register on http://www.clinicaltrials.gov.

## Conclusion

In a randomized, controlled, prospective trial comparing CL and BDUL in patients with DLSS, CL exhibited shorter operation time. In addition, we observed no significant differences in terms of three spine-related pains (LBP, referred buttock pain, and radiating lower leg pain) over time and in terms of functional outcomes (ODI and RMDQ) and SF-36 between the two groups. In single-level decompression of DLSS, group U had the advantages of shorter operation time than group C, but not in terms of back pain, functional outcome, and quality of life.

## Data Availability

All data generated or analyzed during this study are included in this published article.

## Abbreviations

BDUL: Bilateral decompression via unilateral laminotomy
CL: Conventional laminectomy
DLSS: Degenerative lumbar spinal stenosis
LBP: Low back pain
MCS: Mental Component Scale
MRI: Magnetic resonance imaging
ODI: Oswestry Disability Index
PCS: Physical Component Scale
RMDQ: Rolland Morris Disability Questionnaire
VAS: Visual analog scale

## References

[1] Abumi K, Panjabi MM, Kramer KM, Duranceau J, Oxland T, Crisco JJ (1990). Biomechanical evaluation of lumbar spinal stability after graded facetectomies. Spine (Phila Pa 1976)..

[2] Cholewicki J, Panjabi MM, Khachatryan A (1997). Stabilizing function of trunk flexor-extensor muscles around a neutral spine posture. Spine (Phila Pa 1976)..

[3] Tsutsumimoto T, Shimogata M, Ohta H, Misawa H (2009). Mini-open versus conventional open posterior lumbar interbody fusion for the treatment of lumbar degenerative spondylolisthesis: comparison of paraspinal muscle damage and slip reduction. Spine (Phila Pa 1976)..

[4] Rodriguez-Vela J, Lobo-Escolar A, Joven-Aliaga E, Herrera A, Vicente J, Suñén E (2009). Perioperative and short-term advantages of mini-open approach for lumbar spinal fusion. Eur Spine J..

[5] Gille O, Jolivet E, Dousset V, Degrise C, Obeid I, Vital JM (2007). Erector spinae muscle changes on magnetic resonance imaging following lumbar surgery through a posterior approach. Spine (Phila Pa 1976)..

[6] Hara M, Takayasu M, Takagi T, Yoshida J (2001). En block laminoplasty performed with threadwire saw. Neurosurgery..

[7] Kang CH, Shin MJ, Kim SM, Lee SH, Lee CS (2007). MRI of paraspinal muscles in lumbar degenerative kyphosis patients and control patients with chronic low back pain. Clin Radiol..

[8] Kawaguchi Y, Matsui H, Tsuji H (1996). Back muscle injury after posterior lumbar spine surgery. A histologic and enzymatic analysis. Spine (Phila Pa 1976)..

[9] Kim K, Isu T, Sugawara A, Matsumoto R, Isobe M (2008). Comparison of the effect of 3 different approaches to the lumbar spinal canal on postoperative paraspinal muscle damage. Surg Neurol..

[10] Kotil K, Tunckale T, Tatar Z, Koldas M, Kural A, Bilge T (2007). Serum creatine phosphokinase activity and histological changes in the multifidus muscle: a prospective randomized controlled comparative study of discectomy with or without retraction. J Neurosurg Spine..

[11] Liu X, Wang Y, Wu X, Zheng Y, Jia L, Li J (2010). Impact of surgical approaches on the lumbar multifidus muscle: an experimental study using sheep as models. J Neurosurg Spine..

[12] Spetzger U, Bertalanffy H, Reinges MH, Gilsbach JM (1997). Unilateral laminotomy for bilateral decompression of lumbar spinal stenosis. Part II: Clinical experiences. Acta Neurochir (Wien)..

[13] Oertel MF, Ryang YM, Korinth MC, Gilsbach JM, Rohde V (2006). Long-term results of microsurgical treatment of lumbar spinal stenosis by unilateral laminotomy for bilateral decompression. Neurosurgery..

[14] Chang HS, Fujisawa N, Tsuchiya T, Oya S, Matsui T (2014). Degenerative spondylolisthesis does not affect the outcome of unilateral laminotomy with bilateral decompression in patients with lumbar stenosis. Spine (Phila Pa 1976)..

[15] Getty CJ (1980). Lumbar spinal stenosis: the clinical spectrum and the results of operation. J Bone Joint Surg Br..

[16] Deyo RA, Ciol MA, Cherkin DC, Loeser JD, Bigos SJ (1993). Lumbar spinal fusion. A cohort study of complications, reoperations, and resource use in the Medicare population. Spine (Phila Pa 1976)..

[17] Turner JA, Ersek M, Herron L, Haselkorm J, Kent D, Ciol MA (1992). Patient outcomes after lumbar spinal fusions. JAMA..

[18] Liu X, Yuan S, Tian Y (2013). Modified unilateral laminotomy for bilateral decompression for lumbar spinal stenosis. Spine (Phila Pa 1976)..

[19] Hong SW, Choi KY, Ahn Y, Baek OK, Wang JC, Lee SH (2011). A comparison of unilateral and bilateral laminotomies for decompression of L4–L5 spinal stenosis. Spine (Phila Pa 1976)..

[20] Arai Y, Hirai T, Yoshii T, Saka K, Kato T, Enomoto M (2014). A prospective comparative study of 2 minimally invasive decompression procedures for lumbar spinal canal stenosis: unilateral laminotomy for bilateral decompression(ULBD) versus muscle-preserving interlaminar decompression (MILD). Spine (Phila Pa 1976)..

[21] Gejo R, Matsui H, Kawaguchi Y, Ishihara H, Tsuji H (1999). Serial changes in trunk muscle performance after posterior lumbar surgery. Spine (Phila Pa 1976)..

[22] Remes V, Lamberg T, Tervahartiala P, Helenius I, Schlenzka D, Yrjőnen T (2006). Long-term outcome after posterolateral, anterior, and circumferential fusion for high-grade isthmic spondylolisthesis in children and adolescents: magnetic resonance imaging findings after average of 17-year follow-up. Spine (Phila Pa 1976)..

[23] Fan S, Hu Z, Zhao F, Zhao X, Huang Y, Fang X (2010). Multifidus muscle changes and clinical effects of one-level posterior lumbar interbody fusion: minimally invasive procedure versus conventional open approach. Eur Spine J..

[24] Dohzono S, Toyoda H, Matsumura A, Terai H, Suzuki A, Nakamura H (2017). Clinical and radiological outcomes after microscopic bilateral decompression via a unilateral approach for degenerative lumbar disease: minimum 5-year follow up. Asian Spine J..

[25] Nam WD, Chang BS, Lee CK, Cho JH (2014). Clinical and radiological predictive factors to be related with the degree of lumbar back muscle degeneration: difference by gender. Clin Orthop Surg..

